# Neovasculogenic effect of 11,12-epoxyeicosatrienoic acid involves the Akt/eNOS signaling pathways in human endothelial progenitor cells

**DOI:** 10.37796/2211-8039.1343

**Published:** 2022-09-01

**Authors:** Hung-Chang Hung, Jia-Ning Syu, Che-Yi Chao, Shu-Ming Huang, Cheng-Chieh Lin, Mei-Due Yang, Shu-Yao Tsai, Feng-Yao Tang

**Affiliations:** a Department of Internal Medicine, Nantou Hospital, Ministry of Health and Welfare, Nantou City, 540, Taiwan, Republic of China; b Biomedical Science Laboratory, Department of Nutrition, China Medical University, Taichung, 40402, Taiwan, Republic of China; c Department of Food Nutrition and Health Biotechnology, Asia University, Taichung, 41354, Taiwan, Republic of China; d Department of Medical Research, China Medical University Hospital, China Medical University, Taichung, 40402, Taiwan, Republic of China; e Department of Nutrition, Nantou Hospital, Ministry of Health and Welfare, Nantou City, 540, Taiwan, Republic of China; f Department of Nutrition, Master Program of Biomedical Nutrition, Hungkuang University, Taichung City, 43302, Taiwan, Republic of China; g School of Medicine, College of Medicine, China Medical University, Taichung, 40402, Taiwan, Republic of China; h Department of Family Medicine, China Medical University Hospital, Taichung, 40402, Taiwan, Republic of China; i Department of Healthcare Administration, College of Health Science, Asia University, Taichung, 41354, Taiwan, Republic of China; j Department of Surgery, China Medical University Hospital, Taichung, 40402, Taiwan, Republic of China

**Keywords:** 11, 12-Epoxyeicosatrienoic acid, Neovasculogenesis, eNOS, Human endothelial progenitor cells

## Abstract

The 11,12-epoxy-eicosatrienoic acid (11,12-EET) is formed from arachidonic acid (AA) by cytochrome P450 2J2 (CYP 2J2) epoxygenase and function as an effector in blood vessels. Human endothelial progenitor cells (hEPCs), a preceding cell source for endothelial cells (ECs), involve in the vascular tissue repairing by postnatal neovasculogenesis. However, the effect of 11, 12-EET on hEPCs and neovasculogenesis is not well known. In the current study, we examined the function of 11, 12-EET in hEPCs-mediated neovasculogenesis by using tubular formation analysis, Western Blotting assay, immunofluorescence staining, flow cytometry analysis and zymogram analysis. The results suggest that 11, 12-EET significantly induces neovasculogenesis through the phosphorylation of phosphoinositide 3-kinase (PI3–K)/Akt, endothelial-nitric oxide synthase (e-NOS) and extracellular signal-regulated kinase 1/2 (ERK 1/2) signaling pathways. 11, 12-EET up-regulates the expression of cyclin D1, cyclin–dependent kinase 4 (CDK4) and nuclear factor kappa B (NF-κB) proteins. Moreover, 11, 12-EET augments the expression of VE-cadherin and CD31 proteins in hEPCs. 11, 12-EET also augmented Rac1/Rho A signaling cascades, cell migration and an up-regulation of matrix metalloproteinase (MMP) −2 and −9 proteins. These results demonstrate that 11, 12-EET exerts a significant function in the neovasculogenesis of hEPCs.

## 1. Introduction

Bone marrow (BM) provides stem cells and endothelial progenitor cells (hEPCs) for tissue repairment and the prevention of ischemic injuries [1]. Study indicated that human BM-derived hemangioblast, a preceding progenitor for hematopoietic cells, are evolved consequently into hEPCs/endothelial colony forming cells (ECFCs) and endothelial cells (ECs) during embryogenesis and provides the sources for neovascularization [2]. Many studies also suggest that hEPCs are implicated in postnatal neovasculogenesis/angiogenesis process in the adulthood [3,4]. Signaling cascades lead to the neovascularization are involved in the proliferation and outgrowth of hEPCs [5]. Recent studies suggested several important roles of BM-derived circulating hEPCs in neovascularization during different conditions such as wound healing and ischemic injuries [6–8]. Previous studies suggested that the phosphorylation of Akt and extracellular signal-regulated kinase 1/2 (ERK 1/2) proteins induces the proliferation of hEPCs through an increment of nuclear factor kappa B (NF-κB), cyclin D1 and cyclin–dependent kinase 4 (CDK4) proteins [9,10]. The activation of Akt further augments the phosphorylation of downstream targets nuclear factor kappa B inhibitor (IκBα) and endothelial-nitric oxide synthase (e-NOS) proteins to enhance cell migration capability [11]. Moreover, small GTPases such as Rho A and Rac1 proteins are involved in cell movement and angiogenesis through the changes of cytoskeleton, assembly of contractile stress fibers and filopodial/lamellipodial extension [12]. These evidences suggest that RhoA and Rac1 proteins play important roles in cell migration through a modulation of actin cytoskeleton and the stability of microtubules [13,14]. A study also indicated that matrix metalloproteinase (MMP) proteins are involved in the cell recruitment and migration of hEPCs during the neovascularization process [15]. MMP-9 is involved in the augmentation of cell motility of BM-derived progenitor cells [15,16]. Recent study suggests that ECFCs, a late stage of hEPC population, possess the characteristics of a true endothelial progenitor and has a potential to differentiate into ECFCs [17]. A significant up-regulation of CD31 and VE-cadherin was observed during the differentiation of hEPCs into mature ECs [18–20].

A study indicate that CYP2J2 epoxygenase is expressed in vascular ECs and responsible for the conversion of arachidonic acid to the eicosanoid metabolites including 11,12-epoxyeicosatrienoic acids (EETs) [21]. An earlier study suggested that EETs involved in the VEGF-mediated angiogenesis and cancer development [22]. Therefore, the current study would examine the molecular mechanisms of 11, 12-EET in hEPCs-mediated neovasculogenesis.

## 2. Methods

### 2.1. Reagents and antibodies

The matrigel and monoclonal antibody against CD31 (BD 550274) protein were obtained from BD Bioscience (San Jose, CA). The following antibodies were purchased from the CST (Cell Signaling Technology) (Danvers, MA, USA): phosphorylated Akt (p-Akt ^S473^; #4060), total-Akt (t-Akt; #2964), phosphorylated eNOS (p-eNOS ^S1177^; #9571), total-eNOS (t-eNOS; #5880S), phosphorylated ERK 1/2 (p-ERK 1/2 ^T202/Y204^; #9101), total-ERK 1/2 (t-ERK 1/2; #9102), phosphorylated IκBα (p-IκBα ^S32/36^; #9246S), VE-cadherin (#2500S), RhoA (#2117P), phosphorylated Rac1 (p-Rac1 ^S71^; #2461), anti-CDK4 (#2906) and phosphorylated p65 (p-p65/RelA; #3033T). Lamin A (sc-7292), cyclin D1 (sc-8396) and actin (sc-1616) antibodies were purchased from Santa Cruz Biotechnology, Inc (Dallas, TX, USA). MCDB-131 medium, MTT, 11,12-EET (purity >99%), 12-(3-adamantan-1-yl-ureido) dodecanoic acid (AUDA) (soluble epoxide hydrolase inhibitor), PD098059 (MEK inhibitor), Bay-11-7082 (NF-κB inhibitor), wortmannin (PI3–K inhibitor) and l-NAME (eNOS inhibitor) were acquired from Sigma (St Louis, MO). A commercial protein extraction kit, NE-PER, was purchased from Pierce Biotechnology (Lackford, IL). Fetal bovine serum (FBS) was obtained from the Thermo Fisher Scientific (Pittsburgh, PA). EGM-2 growth kit was purchased from Lonza, Inc. (Allendale, NJ). hEPC was a kind gift and provided by Dr. S.C. Chiu (China Medical University, Taichung, Taiwan).

### 2.2. Cell culture procedure

hEPCs were seeded onto culture dish and cultured in MCDB-131 medium with EGM-2 growth kit and 10% fetal bovine serum (FBS). Culture media were changed every 2 days. For these *in vitro* experiments, 11,12-EET was dissolved in dimethyl sulfoxide (DMSO) at a stock solution of 100 mM. hEPCs were treated with 11,12-EET (for 8 h) for further analysis of neovascularization, protein expression, cell migration, proliferation or zymogram analysis.

### 2.3. Assessment of cell survival

hEPCs (2 × 10^4^ cells) were seeded in 24-well plates with MCDB-131 medium containing 11,12-EET and different inhibitors for 8h. MTT assay was used to measure the cell proliferation. At the end of the experiment, the optical absorbance was analysed at wave length of 570 nm with a microplate reader.

### 2.4. Extraction of cellular proteins

Protein extractions were executed by NE-PER kit with inhibitors of phosphatase and protease. To remove the cell debris, cellular proteins were centrifuged for 10 min at 12,000×*g*. The remaining supernatants were obtained as a cytoplasmic fraction.

### 2.5. Western Blotting analysis

Cellular proteins (70 μg) were separated by running in 10% sodium-dodecyl sulfate polyacrylamide gel electrophoresis (SDS-PAGE). The resulting SAS-PAGE gel was electroblotted to polyvinylidene difluoride (PVDF) membrane and covered with primary antibody solution. The blots were stripped and reprobed with internal control antibody.

Detection of other proteins including p-ERK 1/2, p-Akt, p-eNOS, p-IκBα, p-Rac 1 and RhoA was performed by using similar procedure described above. The t-ERK 1/2, t-Akt and t-eNOS antibodies were used as internal controls for p-ERK 1/2, p-Akt, p-eNOS proteins, respectively. Expression of nuclear proteins including cyclin D1, p65/NF-κB (RelA) and CDK4 was measured by also using similar procedure. The Lamin A protein was used as internal control.

### 2.6. Vascular tube formation assay

For the vascular tube formation assay, aliquots of 50 μL matrigel (4 mg/mL) were transferred to each well of a 96-well plate and incubated at 37°C until gelatinization occurred. For the neovasculogenesis assay, hEPCs (1 × 10^4^ cells) were cultured in the matrigel-coated 96 well plate with 10% FBS MCDB-131 medium. After cell seeding for 8 h, photos of neovasculogenesis was documented under inverted phase-contrast microscope. The final results were collected for data analysis by using the accessory software (Olympus imaging system) (Tokyo, Japan).

### 2.7. F-actin assembly detection

hEPCs growing on culture Tek-chamber slides with 11,12-EET (0 and 50 nM) at different time points (0, 0.5 and 2 h) were fixed with phosphate buffer saline (PBS) containing 3.7% formaldehyde and labeled with 5 units/mL of Alexa 488 phalloidin (Invitrogen Inc.). Cells were rinsed three times with PBS and were monitored using Confocal Microscope Detection System (Leica, Wetzlar, Germany) to perform image documentation and analysis.

### 2.8. Flow cytometric analysis of biomarker proteins

hEPCs for biomarker analysis were stained with either anti-human c-kit-fluorescein isothiocyanate (FITC), anti-human CD31-phycoerythrin (PE) or anti-human VE-cadherin-PE solution. Cells stayed on ice for 30 min until analysis by BD FACS Canto flow cytometry (BD Biosciences, Franklin Lakes, NJ). Results from staining cells were analyzed using the accessory software.

### 2.9. Gelatin zymography

Supernatant protein (20 μg) from conditioned medium of cultured hEPCs was transferred into an 8% polyacrylamide gel containing gelatin. At the end of electrophoresis, the polyacrylamide gel was washed with 2.5% Triton X-100 at room temperature and subsequently incubated in a reaction buffer (10 mM CaCl_2_, 0.15 M NaCl and 50 mM Tris) at 37 °C overnight. To detect the enzymatic reaction of MMPs, gel was covered with 0.25% Coomassie blue solution and photograph documented on a light box. Proteolysis within polyacrylamide gel was observed as a white range in a dark field.

### 2.10. Statistical analysis

The biostatistic analysis was performed to investigate the difference in the vascular formation between 11,12-EET groups and control group of hEPCs by using SYSTAT software (Chicago, IL, USA). Confirmation of difference in neovasculogenic index was performed by using the one way ANOVA model and Tukey’s post hoc test at the *P* = 0.05 level. Confirmation of difference in protein expression was performed by using student t-test at the *P* = 0.05 level.

## 3. Results

### 3.1. 11,12-EET significantly induced neovasculogenesis of hEPCs in vitro

**Fig. 1:**
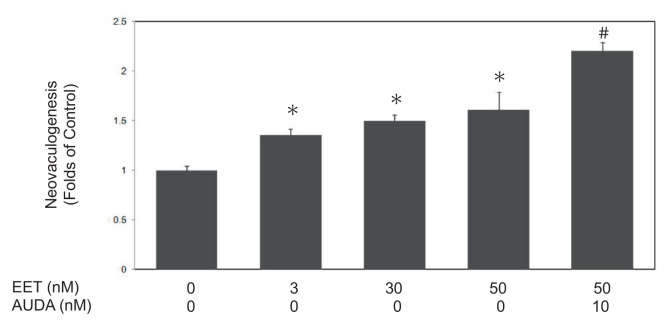
11,12-EET significantly induced neovasculogenesis of hEPCs in vitro. hEPCs were cultured with 11,12-EET (0, 3, 30 and 50 nM) with or without AUDA (a specific inhibitor of sEH) for 8 h until the measurement of neovascularization. These values are presented as mean ± standard deviation (SD) in randomly selected fields in each well. Experiment was performed and reconducted three times. A single asterisk (*) indicates a statistic difference compared to the 11,12-EET-untreated group (P < 0.05). A pound sign (#) indicates a statistic difference compared to the 11,12-EET (50 nM)-treated subgroup (P < 0.05).

In this study, we investigated the effects of 11,12-EET on neovasculogenesis in hEPCs. 11,12-EET (at concentrations of 3, 30 and 50 nM) induced neovascularization of hEPCs by around 1.36, 1.5 and 1.61 folds (*P* < 0.05), respectively (Fig. 1). Treatment of AUDA (at a concentration of 10 nM), an specific inhibitor for soluble epoxide hydrolase (sEH), further enhanced 11,12-EET-mediated neovasculogenesis in hEPCs (*P* < 0.05). These findings indicate that 11,12-EET is involved in the neovasculogenesis of hEPCs.

### 3.2. Akt, eNOS, NF-κB and MAPK/ERK signaling cascades involve in 11,12-eet-mediated neovasculogenesis

**Fig. 2:**
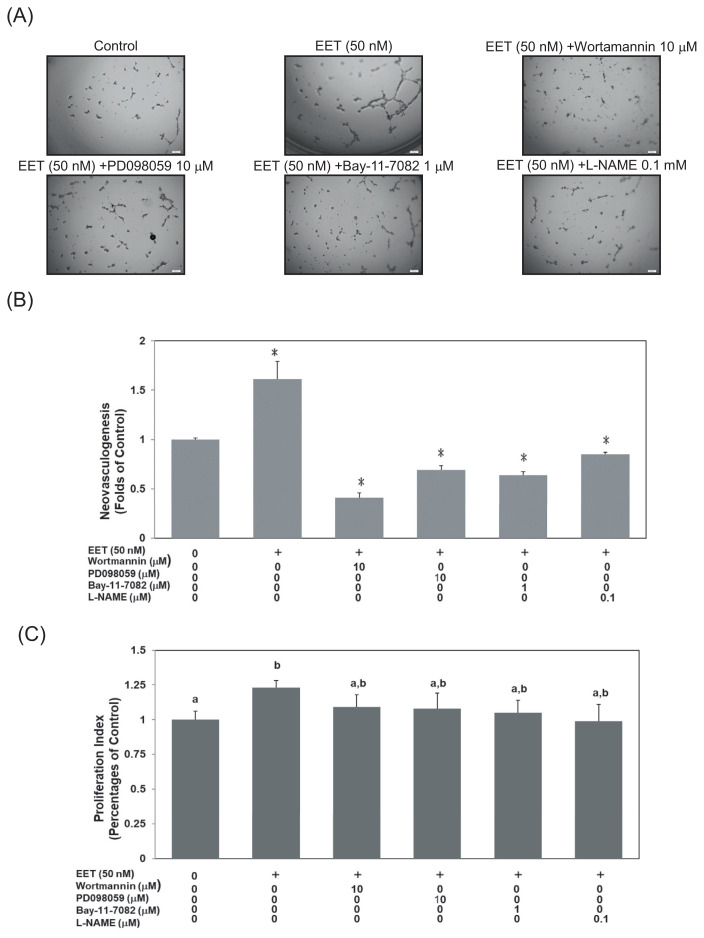
Akt, eNOS, NF-κB and MAPK/ERK signaling cascades involve in 11,12-EET-mediated neovasculogenesis. hEPCs were treated with wortmannin (10 μM), PD098059 (10 μM), Bay-11-7082 (1 μM) and l-NAME (0.1 mM) in the presence of 11,12-EET (50 nM) for 8 h until the analysis of neovasculogenesis (A). The values represent mean ± SD for the quantitative results (B). A single asterisk (*) indicates a statistical difference in comparison with the 11,12-EET-treated group (P < 0.05). Double asterisks (**) indicate a statistical difference in comparison with the 11,12-EET-untreated control group (P < 0.05). Viability analysis of hEPCs was performed under the same treatments. The proliferation index is provided as mean ± SD (C). Different letters represent significant differences of proliferation index among subgroups.

In the current study, we further examined possible signaling pathways in 11,12-EET-mediated neovasculogenesis. Treatment with wortmannin (a specific inhibitor of PI3–K), PD098059 (a specific inhibitor of MEK), Bay-11–7082 (a specific inhibitor of NF-κB) or l-NAME (a specific inhibitor of eNOS) suppressed 11,12-EET-mediated formation of tubular structures in hEPCs, respectively (*P* < 0.05) (Fig. 2 A & B). At a dosage of 10 μM, wortmannin and PD098059 significantly inhibited 11,12-EET-mediated neovascularization up to 75% and 57% respectively. Bay-11-7082 (1 μM) and l-NAME (0.1 mM) each inhibited 11,12-EET-mediated neovascularization up to 60% and 47%. The results also suggested that wortmannin, PD098059, Bay-11-7082 and l-NAME had no cytotoxicity against hEPCs (Fig. 2C). Results suggest that wortmannin, PD098059, Bay-11-7082 and l-NAME exert inhibitory effects on neovasculogenesis without exerting any cytotoxicity. These results indicate that the Akt, eNOS, NF-κB and ERK1/2 molecules are involved in 11,12-EET - mediated neovasculogenesis.

### 3.3. 11,12-EET induced neovasculogenesis through increment of phosphorylated Akt, eNOS and ERK 1/2 proteins in hEPCs

**Fig. 3:**
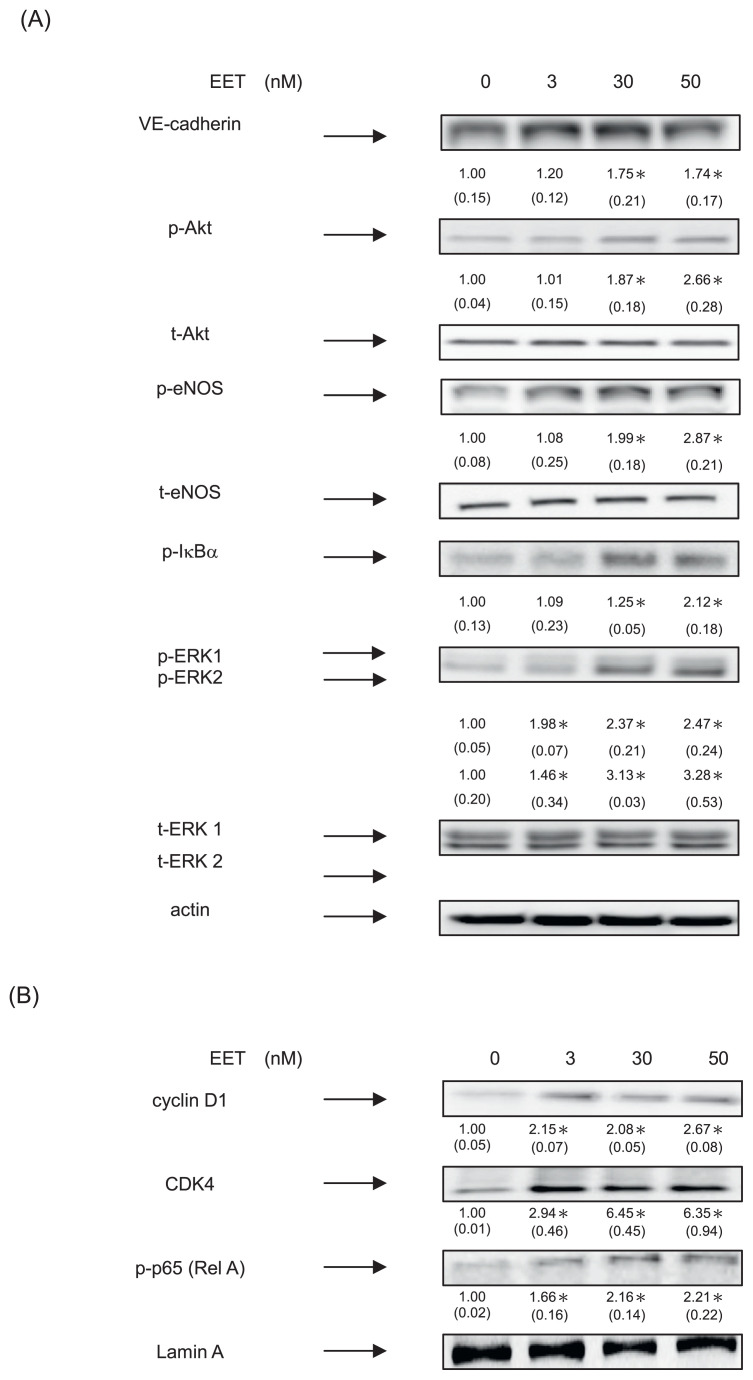
11,12-EET induced neovasculogenesis through increment of phosphorylated Akt, eNOS and ERK 1/2 proteins in hEPCs. hEPCs were treated with 11,12-EET (at concentrations of 0, 3, 30 and 50 nM) for 8 h. (A) Measurement of cytoplasmic proteins including VE-cadherin, p-Akt, t-Akt, p-eNOS, t-eNOS, p-IκBα, p-ERK1/2, t-ERK 1/2 and actin was performed by using Western Blotting analysis as described in Materials and Methods. The integrated densities (mean ± SD) of each protein (VE-cadherin, p-IκBα, p-Akt, p-eNOS, p-ERK1/2) are adjusted with the corresponding control proteins (actin, t-Akt, t-eNOS or t-ERK 1/2) and shown in the bottom row. A single asterisk indicates a statistical difference in comparison with the 11,12-EET untreated control group (P < 0.05). (B) Analysis of nuclear proteins were conducted using antibodies against cyclin D1, CDK4, p-p65 (RelA) and lamin A. The integrated densities (mean ± SD) of these proteins are adjusted with the loading control lamin A protein are shown in the bottom row. A single asterisk represented a statistical difference in comparison with 11,12-EET-untreated control group (P < 0.05).

We further examined whether 11,12-EET would alter the expression of VE-cadherin biomarker protein and the activation of these signaling cascades in hEPCs. 11,12-EET significantly increased the expression of VE-cadherin protein in hEPCs (*P* < 0.05) (Fig. 3A). Moreover, 11,12-EET significantly induced the phosphorylation levels of ERK 1/2, Akt, eNOS and IκBα proteins in hEPCs.

It seems probable, therefore, that 11,12-EET effectively functioned as neovasculogenic agents through increased expression of VE-cadherin proteins as well as the phosphorylation levels of PI3–K/Akt, eNOS, NF-κB, and MAPK/ERK signaling molecules.

To further examine the neovasculogenic effects of 11,12-EET in hEPCs, we measured the expression of nuclear proteins. As shown in Fig. 3B and 11,12-EET enhanced nuclear level of NF-κB (p-p65; RelA) protein in hEPCs. Moreover, 11,12-EET significantly augmented the expression of cyclin D1 and CDK4 proteins in hEPCs. These results suggested that 11,12-EET mediated neovasculogenesis in hEPCs.

### 3.4. 11, 12-EET significantly induced the expression of VE-cadherin and CD31 proteins in hEPCs

**Fig. 4:**
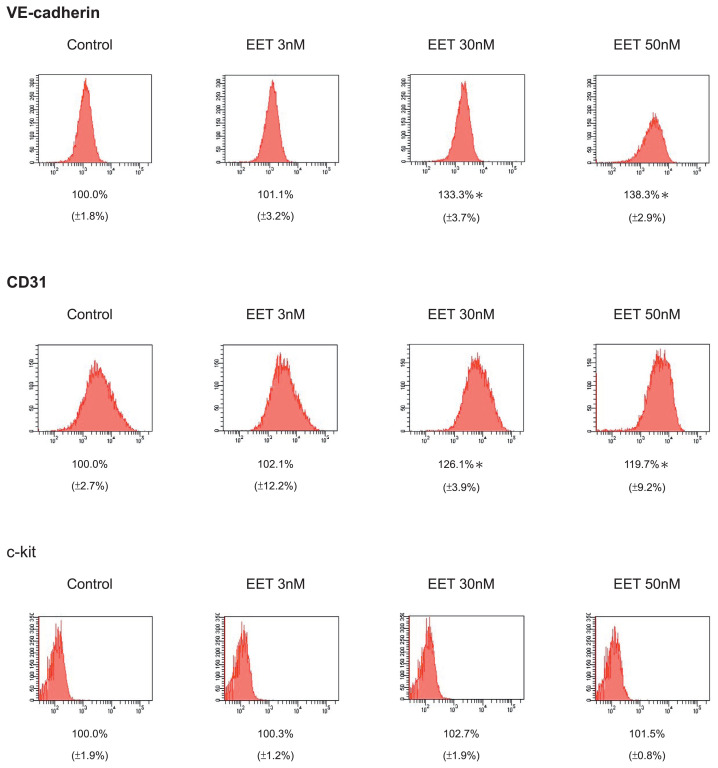
11, 12-EET significantly induced the expression of VE-cadherin and CD31 proteins in hEPCs. The hEPCs were incubated cultured with 11, 12-EET (at concentrations of 0, 3, 30 and 50 nM) in 10% FBS MCDB-131 for 8 h. Detection of VE-cadherin, CD31 and c-kit proteins hEPCs were stained with specific monoclonal antibodies and measured by using flow cytometry analysis as described in Materials and Methods. The amount of detection (mean ± SD) represented the expression level of VE-cadherin and CD31 and c-kit proteins on the surface of h EPCs. Single asterisk represented a statistical difference in comparison with 11,12-EET-untreated control group (P < 0.05).

To further investigate the vasculogenic effects of 11,12-EET in hEPCs, we measured the expression of VE-cadherin, CD31 and c-kit biomarker proteins by using flow cytometry analysis. In Fig. 4, treatment of 11,12-EET significantly increased the VE-cadherin+ and CD-31+ cell subpopulations of hEPCs. These results suggested that EET treatment might augment the differentiation of hEPCs toward to VE-cadherin+/CD31+ late EPCs (ECFCs).

### 3.5. 11,12-EET augmented Rac1/Rho A cascade, cell migration and upregulation of MMP-2,-9 proteins in hEPCs

**Fig. 5:**
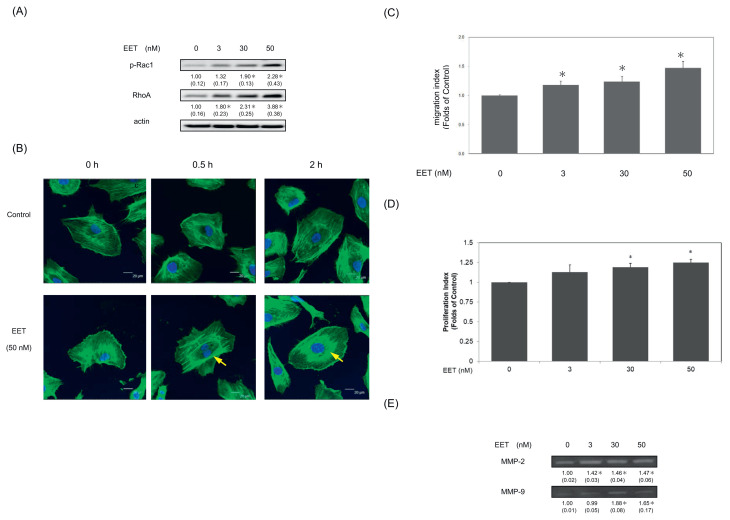
11,12-EET augmented Rac1/Rho A cascade, cell migration and upregulation of MMP-2,-9 proteins in hEPCs. (A) hEPCs were treated with 11,12-EET (0, 3, 30 and 50 nM) in 10% FBS MCDB-131 for 8 h. Measurement of cytoplasmic proteins including p-Rac1, RhoA and actin was performed by using Western Blotting analysis. The integrated densities (mean ± SD) of p-Rac1 and RhoA proteins adjusted with the corresponding loading control protein (actin) are shown in the bottom row. A single asterisk indicates a statistical difference in comparison with the 11,12-EET-untreated control group (P < 0.05). (B) hEPCs were treated in the presence or absence of 11,12-EET (at a concentration of 50 nM) in 10% FBS MCDB-131 at different time points (0, 0.5 and 2 h). F-actin stress fiber were stained with anti-F-actin-FITC antibody using immunofluorescence staining. Imaging was documented at 400× magnification. Green fluorescence indicates the distribution of F-actin. The alignment of F-actin stress fibers was indicated with a yellow arrow. The blue spot represents the distribution of cell nuclei. hEPCs were cultured with 11,12-EET (at concentrations of 3, 30 and 50 nM) for 8 h until the measurement of cell migration (C) and cell proliferation (D). The values are the mean ± SD in 8 randomly selected fields in each culture well, each carried out in triplicate and repeated twice. A single asterisk (*) indicates a statistically significant difference compared to the 11,12-EET-untreated control group (P < 0.05). (E) hEPCs were treated with 11,12-EET (0, 3, 30 and 50 nM) in MCDB-131 for 8 h. Conditioned media were collected for zymogram analysis. The levels of detection represented the expression of MMP-2 and MMP-9 proteins secreted from hEPCs. The densities (mean ± SD) of these proteins are shown in the bottom row. A single asterisk represents a significant difference of MMP-2 or MMP-9 expression in comparison to the 11,12-EET-untreated group (P < 0.05).

To verify actions of 11, 12-EET on cell migration, we further elucidated the probable effects of 11,12-EET on Rac 1 and Rho A proteins. As demonstrated in Fig. 5A and 11,12-EET increased phosphorylated levels (i.e. activation) of the RAC 1 and an upregulation of Rho A proteins. The results suggested that 11,12-EET could modulate cell migration and enhance the expression of phosphorylated-Rac 1 and Rho A proteins in hEPCs.

To confirm these findings, we further examined whether 11,12-EET altered the distribution of F-actin proteins in hEPCs. As shown in Fig. 5B, F-actin stress fibers is randomly distributed in hEPCs at starting time point (Fig. 5B, a-b). Treatment of 11, 12-EET (50 nM) significantly induced the redistribution of F-actin stress fibers into a well-aligned pattern in hEPCs (Fig. 5B, c-f). 11,12-EET also significantly induces cell migration of hEPCs (*P* < 0.05) (Fig. 5C). Our results demonstrated that 11,12-EET increased cell proliferation levels in hEPCs (Fig. 5D). Previous study suggested that neovasculogenesis and angiogenesis are correlated with an increasing enzymatic activity of MMP-2 and MMP-9 proteins. Therefore, we further investigated whether 11,12-EET modulated MMP-2 and MMP-9 expression in hEPCs. In Fig. 5E, 11,12-EET effectively augmented the enzymatic activities of both MMP-2 and MMP-9 (P < 0.05). The results suggested that 11,12-EET exerted neovasculogenesis activity and induced the breakdown of extracellular matrix through an upregulation of MMP-2 and MMP-9 proteins in hEPCs.

## 4. Discussion

Many studies already showed that neovasculogenesis and angiogenesis play key steps in the physiological and pathological conditions including ischemia prevention and tumor development [23]. Moreover, studies suggested important roles of EETs in cardioprotection, angiogenesis and tumor metastasis [22,24,25]. Therefore, EETs seem to play important roles in many aspects of chronic diseases. Adult neovasculogenesis occur through the circulating hEPCs into vascular sites and the proliferation of ECs [26–28]. A recent study suggested that well-differentiated ECs with a low proliferative potential have low repairing capability for damaged ECs. hEPCs from adult BM obtain an early-onset differentiated capacity, an excellent repairing capability and neovasculogenesis-prone characteristics.

Many studies suggested that EETs generated by cytochrome P450 epoxygenases are vasodilators eicosanoids [29,30]. These P450 epoxygenases CYP2J2 are highly expressed in vascular ECs and capable of metabolizing arachidonic acid into several types of EETs including 11,12-EET [21]. Previous studies showed that a risk of CAD is inversely correlated with the levels of the Cytochrome P450 epoxygenases CYP2J2 and 11,12 EET [31,32]. EETs are also involved in the VEGF-mediated angiogenesis processes [22]. These results suggested that 11,12-EET has favorable effects on increasing endothelial functions, neovasculogenesis and even angiogenesis. A recent study indicated that 11,12-EET could promote organ and tissue regeneration [33]. Moreover, 11,12-EET could rescue impaired wound healing under ischemic condition [34]. These *in vivo* findings suggested that 11,12-EET could act as an effective agent to increase neovascularization and prevent ischemic injuries.

For the first time, we demonstrated that 11,12-EET induces neovascularization/angiogenesis through an EPC-differentiation into EC-like characteristics. The results showed that 11,12-EET significantly augmented neovasculogenesis of hEPCs, in part, through an up-regulation of VE-cadherin protein *in vitro*. The molecular actions of 11,12-EET were mediated through the activation of signaling pathways including Akt, eNOS and ERK 1/2 signaling cascades. 11,12-EETalso augmented the expression of RhoA and the phosphorylation of Rac 1 proteins. These novel findings suggested that 11,12-EET may function as a signaling effecter to induce neovasculogenesis.

The results further demonstrated that 11,12-EET significantly increased the nuclear levels of cyclin D1 and CDK4 proteins in hEPCs. There are several possible scenarios for 11,12-EET to induce neovasculogenesis and angiogenesis. One explanation might be predominant activation of Akt, eNOS and MAPK/ERK molecules in hEPCs. Neovascularization requires the activation of Akt and ERK 1/2 pathways and eNOS protein in hEPCs. The Akt-mediated phosphorylation of eNOS would lead to an increasing cell migration of endothelial cells [35]. Inhibition of the signaling cascades including PI3–K/Akt and eNOS pathways was also correlated with increased apoptosis level in ECs [35]. Our results suggested that 11,12-EET plays an important role by induction of NO bioavailability in vascular tissues. Although we have not examined the stability of 11,12-EET and the metabolic consequence, it is probable that 11,12-EET obtains its effect on neovascularization.

Alternatively, we identify a new role of 11,12-EET in the upregulation of cyclin D1 and CDK4 proteins in hEPCs. It is probable that a closely correlation between the expression of cyclin D1 and CDK4 proteins in 11, 12-EET mediated neovasculogenesis. Results from the current study further implicated that 11,12-EET probably induced the differentiation of hEPCs into ECFCs (late-EPCs) through increased levels of VE-cadherin and CD31 proteins. Based on our observation, it is probable that 11,12-EET may function as an agent to induce differentiation of EPC into ECFCs. After all, 11,12-EET induced the differentiation of EPC into ECFCs and the proliferation of these cells through augmented expression of cyclinD1 and CDK4 proteins as well as the neovasculogenesis in hEPCs. These results prove the neovasculogenic and angiogenic effects of 11,12-EET. It may explain the important roles of 11,12-EET in the differentiation of BM-derived hEPCs and the proliferation of ECFCs.

To validate these important findings, we also measured the expression of MMP-2 and MMP-9 proteins in these 11,12 EET-treated EPCs. As shown in Fig. 5, these results suggested that 11,12-EET-mediated cell migration and neovascularization were associated with augmented expression of MMP-2 and MMP-9 proteins in hEPCs. Augmented expression of MMP proteins and the activation of Rac 1 and RhoA molecules are observed in the 11,12 - EET-mediated neovasculogenesis. Although we have not verified the crucial role of 11,12-EET in experimental animal model yet, the probable limitation is to measure the bioavailability of 11,12-EET in an *in vivo* study. Previous studies indicated that increased activities of endothelial progenitor cells could play important roles in increment of wound healing, tissue repairment and tissue regeneration [1]. One of probable utilization of 11,12-EET could be applied to promote wound healing or tissue repairment in future clinical application.

## 5. Conclusion

**Fig. 6:**
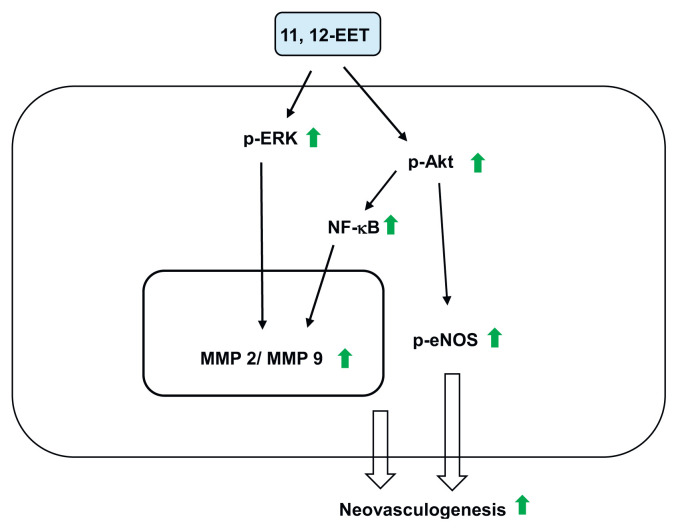
Proposed mechanisms of 11, 12-EET-mediated neovasculogenesis in hEPCs. Green arrow indicates an increased level.

In conclusion, the specific novel aspects of this study include the neovasculogenic effects of 11,12-EET on (i) the activation of PI3–K/Akt, eNOS and ERK 1/2 signaling pathways; (ii) the differentiation of EPCs into ECFCs; (iii) the proliferation of EPCs; and (iv) the cell migration of EPCs. The proposed mechanism was described in Fig. 6. Our results might provide an important insight leading to the application of 11,12-EET in the future preclinical and clinical studies.

## Supplementary Information
